# Post-Diagnostic Lifestyle Adaptations in Fibromyalgia: A Network and Cluster Analysis of Real-World Behavioral Patterns

**DOI:** 10.3390/nu18111791

**Published:** 2026-06-02

**Authors:** Matylda Kosiorz, Małgorzata Muc-Wierzgoń, Katarzyna Weronika Walkiewicz, Sylwia Dzięgielewska-Gęsiak

**Affiliations:** 1 Student Scientific Association at the Department of Internal Medicine and Emergency Medicine, Medical University of Silesia in Katowice, 41-902 Katowice, Poland; s84754@365.sum.edu.pl; 2 Department of Propedeutics of Internal Medicine and Emergency Medicine, Medical University of Silesia in Katowice, 41-902 Katowice, Polandsgesiak@sum.edu.pl (S.D.-G.)

**Keywords:** musculoskeletal disease, fibromyalgia, lifestyle adaptation, dietary modification, physical activity, behavioral patterns, cluster analysis

## Abstract

Background: Fibromyalgia is a chronic pain disorder. Patients often introduce lifestyle changes, including dietary modifications and physical activity, after diagnosis to alleviate symptoms. However, real-world patterns of these post-diagnostic adaptations remain poorly understood. Methods: An exploratory cross-sectional study was conducted in 88 patients with fibromyalgia using a study-specific questionnaire. Spearman correlation and network analysis were applied to assess relationships between lifestyle changes. K-means clustering was used to identify adaptation patterns. Differences in symptom severity were evaluated using the Kruskal–Wallis test and regression analysis. Results: Participants most frequently increased vegetable and water intake and reduced consumption of meat, alcohol, and sugar-sweetened beverages. Network analysis revealed structured co-occurrence patterns among dietary changes. Three clusters were identified: minimal (n = 47), selective (n = 27), and comprehensive (n = 14). No significant association was found between adaptation patterns and symptom severity (*p* = 0.53). Conclusions: Patients with fibromyalgia may adopt structured, non-random lifestyle changes following diagnosis. These findings provide a data-driven perspective on real-world behavioral adaptation patterns and highlight the need for longitudinal research to better understand their potential role in disease management.

## 1. Introduction

Fibromyalgia (FM) is a chronic multifactorial disorder characterized by widespread pain, fatigue, sleep disturbances, and cognitive and emotional symptoms, leading to substantial impairment in daily functioning [1,2,3]. According to the International Association for the Study of Pain (IASP) classification for ICD-11, fibromyalgia is categorized as chronic primary widespread pain [4]. The global prevalence is estimated at approximately 2–4% of the adult population, with a higher incidence observed in women [5,6,7].

The pathophysiology of fibromyalgia is still not fully understood and is considered multifactorial, involving central sensitization, altered pain processing, neuroendocrine dysregulation, and changes in neurotransmitter activity [8,9,10,11]. These complex mechanisms contribute to heightened pain sensitivity and variability in symptom presentation, supporting the view of fibromyalgia as a heterogeneous condition [12,13,14,15,16,17].

Current management of fibromyalgia is based on a multidisciplinary approach combining pharmacological and non-pharmacological strategies. Among non-pharmacological interventions, lifestyle-related factors—including physical activity, diet, and behavioral modifications—play a central role in symptom management and overall quality of life [3,18,19]. Regular physical activity, particularly aerobic and resistance exercise, is strongly recommended in clinical guidelines and has been shown to improve pain, fatigue, and functional capacity [20,21].

In contrast, dietary recommendations for fibromyalgia remain less clearly defined. Various dietary approaches—including Mediterranean, low-FODMAP, vegetarian, and elimination diets—have been investigated, but the available evidence is heterogeneous and does not support a single standardized nutritional strategy [22,23,24,25,26]. Nevertheless, diet is increasingly recognized as a potentially modifiable factor influencing inflammation, metabolic pathways, and symptom perception in chronic pain conditions.

In clinical practice, many patients independently introduce lifestyle modifications following diagnosis, often without structured guidance. These changes may include alterations in dietary habits, physical activity, and other health-related behaviors aimed at symptom relief and improved well-being [19,27,28,29,30]. However, most existing studies focus on controlled interventions, while spontaneous, real-world adaptation patterns remain poorly understood.

Understanding how patients naturally modify their lifestyle following diagnosis may provide valuable insight into behavioral responses to chronic disease. Importantly, such adaptations may not occur as isolated changes but rather as structured and interrelated patterns of behavior. Such an approach may contribute to a better understanding of behavioral heterogeneity in fibromyalgia while complementing symptom-based classifications.

However, it remains unclear whether these post-diagnostic changes occur randomly or instead form consistent and identifiable behavioral patterns across individuals.

We hypothesized that post-diagnostic lifestyle modifications are structured and interrelated and can be grouped into distinct adaptation profiles. Accordingly, this study aimed to characterize the direction and co-occurrence of dietary and physical activity changes, identify behavioral adaptation patterns using network and cluster analyses, and explore their cross-sectional relationship with symptom severity.

## 2. Materials and Methods

### 2.1. Ethics

According to national regulations, this study did not meet the criteria of a medical experiment and did not require approval from a bioethics committee. Nevertheless, the study was conducted in accordance with the principles of the Declaration of Helsinki (2013). Participation in the study was voluntary and based on informed consent.

Respondents’ data were pseudonymized to ensure confidentiality. Personal data were processed in such a way that they could not be attributed to a specific individual without the use of an additional identification key. The survey was conducted anonymously, and no directly identifiable personal data were collected.

### 2.2. Study Design and Participants

This exploratory, cross-sectional study was designed to investigate post-diagnostic lifestyle adaptations in a real-world population of patients with fibromyalgia. A total of 96 individuals with a self-reported diagnosis of fibromyalgia were recruited and invited to complete the questionnaire. After exclusion of incomplete responses, 88 participants were included in the final analysis.

Inclusion criteria were a self-reported medical diagnosis of fibromyalgia and completion of the questionnaire. Exclusion criteria comprised incomplete questionnaires or conditions that could substantially interfere with the assessment of lifestyle behaviors.

The study was conducted using a study-specific, self-administered questionnaire developed to assess post-diagnostic lifestyle adaptations in patients with fibromyalgia.

The questionnaire included 26 items covering demographic characteristics, disease duration, comorbidities, clinical symptoms, physical activity, and dietary habits. A key component of the instrument was the assessment of changes in lifestyle behaviors following diagnosis. Participants were asked to indicate whether the consumption of specific food items or engagement in physical activity had increased, decreased, or remained unchanged.

A test–retest procedure with a 30-day interval was conducted in 20 individuals to assess questionnaire clarity and response reproducibility. Formal reliability coefficients (e.g., Cohen’s kappa or intraclass correlation coefficients) were not calculated during the pilot phase, representing a methodological limitation. The pilot assessment was primarily intended to identify issues related to item clarity and response stability prior to the main study.

The questionnaire was specifically designed to capture the directionality of behavioral changes rather than to quantify absolute levels of dietary intake or physical activity. Importantly, the use of simplified categorical responses facilitated consistent recall of post-diagnostic changes, which may be more reliable than retrospectively quantifying absolute intake levels. For this reason, standardized validated instruments (e.g., FIQ-R or IPAQ) were not directly applicable to the primary research objective.

Although full psychometric validation was not performed, the instrument was developed on the basis of existing literature and expert-informed content and was considered appropriate for exploratory identification of behavioral adaptation patterns in a real-world setting.

### 2.3. Assessment of Physical Activity

Physical activity was assessed using a simplified self-reported classification intended to capture general behavioral tendencies rather than precise activity volume. This approach was chosen to reduce respondent burden and facilitate recall within a retrospective survey design. Physical activity variables were used primarily for descriptive purposes and were not included in the clustering procedure.

Light physical activity corresponded to irregular or low levels of exercise (<150 min/week), whereas moderate activity reflected regular physical activity consistent with general health recommendations (approximately 150 min/week). High and very high activity levels referred to more intensive or more frequent physical exercise exceeding these thresholds [31,32]. Participants also reported the types of physical activity they performed, including walking, Nordic walking, yoga, cycling, and swimming.

### 2.4. Statistical Analysis

Descriptive statistics were calculated to summarize participant characteristics and reported lifestyle changes following fibromyalgia diagnosis.

The analytical approach was exploratory and aimed to identify patterns of co-occurring behavioral changes rather than establish causal relationships.

To enable quantitative analysis of lifestyle modifications, responses were recoded into ordinal variables: +1 for an increase, 0 for no change or no prior consumption, and −1 for a decrease. This recoding was designed to capture the directionality of behavioral changes, which was central to the research objective. Although this approach does not reflect the magnitude of changes, it allows for the identification of structured co-occurrence patterns between behaviors.

Associations between behavioral modifications were assessed using Spearman’s rank correlation coefficients. Based on statistically significant correlations (*p* < 0.05, |ρ| ≥ 0.25), a network analysis was conducted to explore structural relationships between lifestyle adaptations.

No formal network stability analyses (e.g., bootstrapping of centrality estimates) were conducted due to the exploratory nature of the study and relatively small sample size. Accordingly, network centrality findings should be interpreted as descriptive and hypothesis-generating rather than confirmatory.

To identify distinct behavioral adaptation patterns, k-means cluster analysis was performed using dietary variables representing the direction of reported changes. Cluster analysis was applied as a data-driven approach to group participants with similar behavioral profiles. Physical activity variables were used for descriptive characterization of clusters but were not included in the clustering procedure.

The clustering solution (k = 3) was selected on the basis of interpretability, cluster separation, and inspection of within-cluster variance. Given the relatively small sample size and exploratory design, the identified clusters should be interpreted as hypothesis-generating patterns rather than as stable clinical phenotypes.

Differences in symptom severity across clusters were assessed using the Kruskal–Wallis test. Additionally, linear regression analysis adjusted for age and time since diagnosis was performed to explore the cross-sectional relationship between adaptation patterns and symptom severity.

Statistical significance was set at *p* < 0.05. All analyses were conducted using Statistica 13.3.

## 3. Results

### 3.1. Baseline Characteristics and Physical Activity

A total of 96 individuals with fibromyalgia were recruited, of whom 88 respondents provided complete data on the main study variables and were included in the final analysis. The cohort was predominantly female (95.5%), with a mean age of 46.9 ± 11.3 years and a mean BMI of 25.6 ± 4.6 kg/m^2^.

Participants represented a broad range of disease duration, including both recently diagnosed individuals and those with long-standing fibromyalgia.

The mean Widespread Pain Index (WPI) score was 16.7 ± 7.0, and the mean Symptom Severity Scale (SSS) score was 6.1 ± 1.8, indicating a moderate symptom burden. Baseline characteristics are summarized in Table 1.

The heterogeneity in disease duration enabled the exploration of behavioral adaptations across different stages of the disease. However, the study did not assess whether dietary and physical activity strategies evolved during the course of the disease.

**Figure 1:**
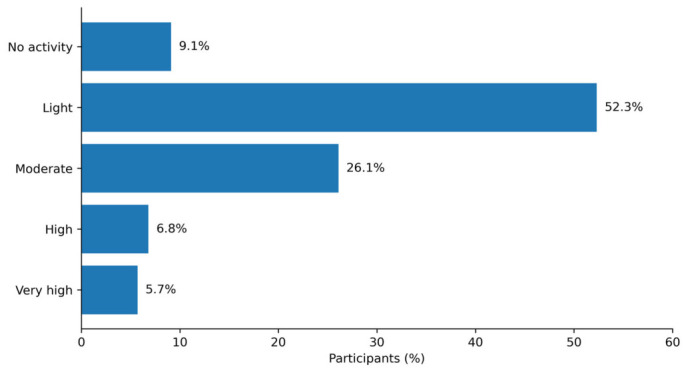
Distribution of self-reported physical activity levels among patients with fibromyalgia (N = 88). Horizontal bar chart presenting the percentage distribution of self-reported physical activity categories among study participants. Physical activity levels were classified as no activity, light, moderate, high, or very high based on self-reported frequency and intensity of exercise. Percentages were calculated for the total study population.

Data on physical activity were available for 88 patients. More than half of the respondents reported light physical activity (52.3%), while 26.1% declared moderate activity levels. No physical activity was reported by 9.1% of participants, whereas high and very high levels were relatively uncommon (6.8% and 5.7%, respectively) (Figure 1).

With regard to frequency, the most common response was engaging in physical activity several times per week (38.5%). Daily activity was reported by 18.8% of participants, while 11.5% exercised once per week and 11.5% several times per month. A small proportion of participants reported only occasional activity. The most frequently reported form of activity was walking (76.1%), followed by yoga (29.5%), cycling (15.9%), Nordic walking (11.4%), and swimming (8.0%). No participants reported aerobics or Pilates. Because multiple responses were allowed, percentages do not sum to 100% (Table 2).

### 3.2. Direction and Frequency of Post-Diagnostic Dietary Modifications

Descriptive analysis indicated that dietary modifications following fibromyalgia diagnosis were common, although both the direction and magnitude of changes varied among participants. The most frequently reported health-promoting modifications included increased consumption of vegetables (n = 36) and water (n = 41). Reductions were most commonly observed for meat (n = 36), alcohol (n = 30), and sugar-sweetened beverages such as cola (n = 36). A substantial proportion of participants reported no prior consumption of certain products, particularly alcohol (22.7%) and sugar-sweetened beverages (20.5%), which may have influenced the observed patterns of change (Table 3).

It should be noted that these values refer to the total study population (N = 88), including individuals who reported no prior consumption of specific products.

Only statistically significant correlations meeting the predefined threshold (*p* < 0.05, |ρ| ≥ 0.25) are presented. These significant correlations between dietary changes reflect the co-occurrence of specific behaviors.

**Figure 2:**
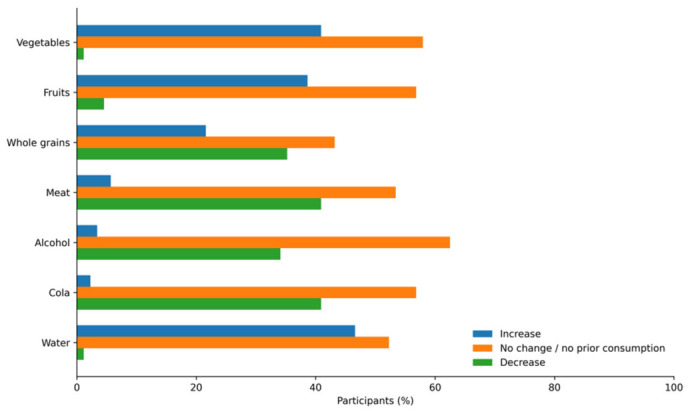
Reported dietary changes after fibromyalgia diagnosis.

The distribution of reported dietary changes introduced after diagnosis is presented in Figure 2.

Grouped horizontal bar chart presenting the percentage distribution of self-reported dietary behavior changes after diagnosis. Dietary modifications were categorized as increased consumption, decreased consumption, or no change/no prior consumption. Percentages were calculated for the total study population (N = 88).

The distribution of reported dietary changes introduced after diagnosis is presented in Figure 2. To investigate the co-occurrence and structural organization of these behavioral modifications, Spearman correlation analysis was performed. Several statistically significant associations between dietary behavior changes were identified (*p* < 0.05, |ρ| ≥ 0.25) (Table 3).

Increased vegetable consumption was positively correlated with increased water intake (ρ = 0.32). Reductions in sugar-sweetened beverage consumption (cola) were moderately correlated with reductions in alcohol intake (ρ = 0.41).

Additionally, increased vegetable intake was associated with reduced consumption of cola (ρ = 0.28) and alcohol (ρ = 0.26).

These findings indicate that lifestyle changes following fibromyalgia diagnosis tend to co-occur and form structured behavioral patterns rather than occurring independently.

### 3.3. Network Structure of Dietary and Physical Activity Adaptations

**Figure 3:**
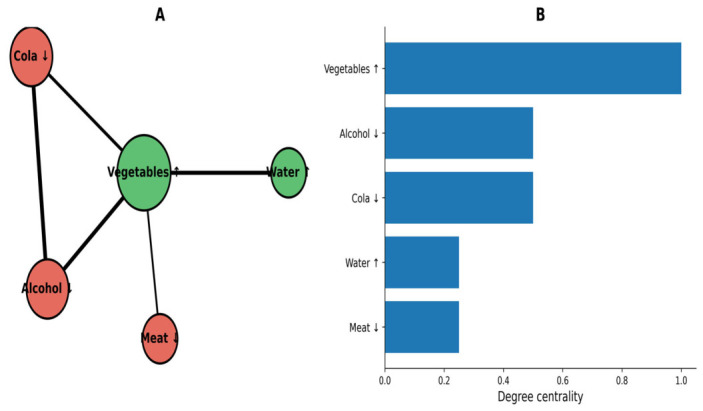
Network structure of dietary behavior changes following fibromyalgia diagnosis: (**A**) Network visualization of significant co-occurring dietary behavior changes. (**B**) Degree centrality analysis of dietary behavior changes within the network. ↑ indicates an increase; ↓ indicates a decrease.

To further examine the structural organization of behavioral changes, a network analysis was conducted using dietary behavior changes as nodes and statistically significant Spearman correlations (*p* < 0.05, |ρ| ≥ 0.25) as edges (Figure 3A,B).

The network exhibited a connected structure, indicating interrelationships among dietary behavior changes. Nodes represent individual dietary changes, with color indicating direction (green = increase, red = decrease) and node size proportional to degree centrality. Edges represent statistically significant co-occurrence between behaviors.

Degree centrality analysis showed that increased fruit consumption and reduced meat and alcohol intake had the highest centrality values (DC = 0.67), followed by increased vegetable intake (DC = 0.50), while whole-grain intake demonstrated lower centrality (DC = 0.17).

**Figure 4:**
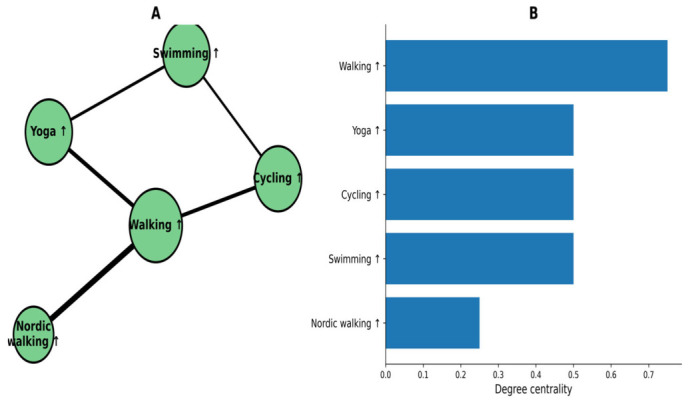
Network structure of physical activity adaptations following fibromyalgia diagnosis: (**A**) Network visualization of co-occurring physical activity behaviors. (**B**) Degree centrality analysis of physical activity behaviors within the network. ↑ indicates an increase.

The network of physical activity changes demonstrated a centralized structure, with walking-based activities emerging as the most central node. Other activity types, such as yoga and cycling, showed lower centrality, whereas swimming and physical inactivity remained peripheral (Figure 4A,B).

Nodes represent types of physical activity, with color indicating the direction of change (green = increase, red = decrease), and node size proportional to centrality. Edges represent the co-occurrence of reported changes between activity types.

Walking-based activities, including walking and Nordic walking, formed the central component of the network, whereas other activity types showed lower connectivity and centrality values.

### 3.4. Cluster Analysis of Dietary Adaptation Patterns

Cluster analysis identified three distinct dietary adaptation patterns among patients with fibromyalgia. The minimal adaptation pattern was the most common (n = 47), followed by the selective dietary modification pattern (n = 27), while the comprehensive health-oriented pattern was the least frequent (n = 14).

Participants in the comprehensive pattern reported multiple dietary changes, including increased consumption of vegetables, fruits, whole grains, and water, together with reduced intake of meat, alcohol, and sugar-sweetened beverages. The selective pattern was characterized by targeted modifications, primarily involving increased consumption of plant-based foods and a partial reduction in the consumption of selected products. The minimal pattern was characterized by limited or inconsistent changes across dietary variables.

Mean symptom severity scores (WPI + SSS) were similar across clusters, with values of 23.21, 22.92, and 22.80 for the comprehensive, selective, and minimal patterns, respectively. No statistically significant differences in symptom severity were observed between clusters (*p* = 0.53) (Table 4).

Distinct behavioral adaptation patterns were identified; however, no statistically significant association between the extent of lifestyle modification and symptom severity was observed in this cohort.

**Figure 5:**
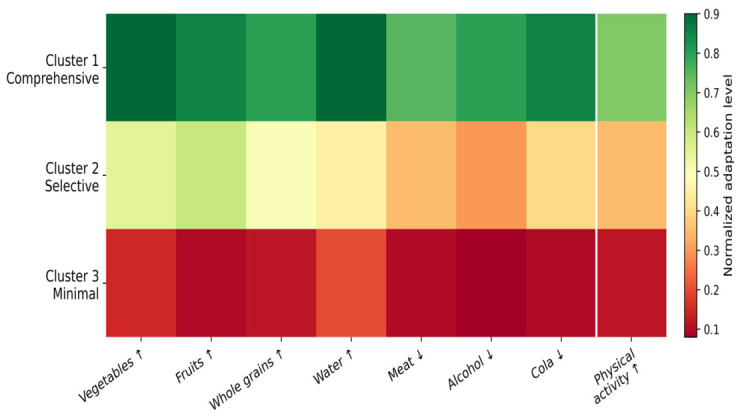
Behavioral profiles of dietary and lifestyle adaptation patterns identified by cluster analysis. Heatmap presenting normalized behavioral adaptation profiles across the identified clusters. Green indicates higher levels of pro-health adaptation, whereas red indicates lower levels or the absence of adaptation. Cluster 1 represents the comprehensive health-oriented pattern, Cluster 2 the selective dietary modification pattern, and Cluster 3 the minimal adaptation pattern. ↑ indicates an increase; ↓ indicates a decrease.

The profiles of the identified clusters are shown in Figure 5.

Cluster 1—Comprehensive health-oriented pattern

This group demonstrated the highest levels of health-promoting dietary change and greater engagement in physical activity, particularly low-impact forms such as walking and yoga, indicating a broad lifestyle-oriented adaptation.

Cluster 2—Selective dietary modification pattern

Participants exhibited targeted dietary changes, mainly increased intake of plant-based foods and whole grains, with less consistent modifications in other domains.

Cluster 3—Minimal adaptation pattern

This cluster was characterized by limited or inconsistent changes across most variables, including lower levels of physical activity, suggesting the absence of a coherent behavioral adaptation strategy.

### 3.5. Association Between Adaptation Patterns and Symptom Severity

Differences in symptom severity (WPI + SSS) across clusters were assessed using the Kruskal–Wallis test, which showed no significant differences (*p* = 0.53). In multivariable linear regression analysis adjusted for age and time since diagnosis, the Adaptation Index was not associated with symptom severity (*p* = 0.83). The model explained a small proportion of the variance (R^2^ = 0.064).

These findings indicate that, despite the presence of structured behavioral adaptation patterns, their cross-sectional association with symptom severity appears limited.

## 4. Discussion

To our knowledge, this is one of the first studies to apply a combined network and cluster-analytic approach to investigate real-world lifestyle adaptations in patients with fibromyalgia, thereby providing a data-driven perspective on behavioral pattern formation.

The findings suggest that lifestyle changes introduced after diagnosis are not random but instead form structured and interrelated patterns. Using network analysis and clustering, three distinct adaptation profiles—minimal, selective, and comprehensive—were identified, providing a novel perspective on patient-driven behavioral responses in fibromyalgia that extends beyond traditional intervention-based approaches. Most previous studies on fibromyalgia have focused on non-pharmacological interventions—such as specific diets, structured exercise programs, patient education, and cognitive–behavioral therapy—as complementary approaches aimed at improving clinical outcomes [18,33,34,35,36]. In contrast, the present study captures spontaneous, real-world adaptations implemented by patients in everyday settings. The observed clustering of behaviors suggests that patients tend to adopt coherent strategies rather than isolated changes. For example, increased consumption of vegetables and water co-occurred with reduced intake of alcohol and sugar-sweetened beverages, consistent with a shift toward healthier dietary patterns.

Importantly, the identified adaptation patterns may reflect distinct behavioral adaptation profiles in fibromyalgia. This perspective aligns with the growing recognition that fibromyalgia is a heterogeneous condition, not only in terms of symptom presentation but also with regard to patient coping strategies and health-related behaviors [8,9,37]. Recent evidence highlights that behavioral patterns, including avoidance, overactivity, and pacing, play a central role in shaping symptom burden and daily functioning in fibromyalgia [38,39,40,41,42].

In the present study, the distinction between minimal, selective, and comprehensive adaptation patterns suggests that patients may follow different trajectories of behavioral response following diagnosis. These trajectories may be influenced by factors such as health beliefs, symptom perception, prior experiences, and access to health information, although these determinants were not directly assessed. Future studies should examine how these patterns evolve over time and whether they are associated with changes in disease activity or other clinically relevant outcomes [43].

Dietary changes were most frequently reported within the first three years following diagnosis, suggesting that this period may represent a critical window for behavioral adaptation. This observation is consistent with the concept of a “teachable moment” in chronic disease, during which individuals may be more receptive to health-related behavior change [43,44].

Despite the presence of distinct adaptation patterns, no association was observed between the extent of lifestyle modification and symptom severity. However, this finding should be interpreted with caution, given the cross-sectional design of the study. Lifestyle modifications were reported retrospectively as changes introduced after diagnosis, whereas symptom severity was assessed at the time of survey completion. Therefore, the observed lack of association may have been influenced by symptom severity at the time of diagnosis, which was not assessed in this study. Patients with more severe symptoms earlier in the disease course may have been more likely to implement lifestyle modifications, while current symptom burden may reflect multiple subsequent factors.

Consistent with previous literature, dietary approaches in fibromyalgia remain heterogeneous and often show inconsistent effects on symptom severity. In contrast, the present findings suggest that patients tend to adopt gradual and selective dietary modifications rather than follow structured dietary protocols. This may indicate that real-world dietary adaptation is more flexible and individualized than interventions evaluated in controlled clinical settings [45,46,47,48,49,50,51,52].

The physical activity patterns observed in this study were partially consistent with current recommendations. Walking and other low-intensity activities were the most commonly reported forms of exercise, which may reflect patients’ preference for accessible and tolerable forms of movement in this population. The central role of walking within the behavioral network further underscores its prominence among reported activity patterns. However, current recommendations extend beyond low-intensity aerobic activity. Both the EULAR recommendations for fibromyalgia management and the World Health Organization guidelines emphasize the importance of regular aerobic exercise combined with muscle-strengthening or resistance training [31,32]. The relatively low frequency of resistance-based activities in our cohort may suggest that patients preferentially choose more accessible forms of exercise while underutilizing other guideline-recommended modalities.

Several limitations of this study should be acknowledged. First, the study combined retrospectively reported lifestyle changes introduced after diagnosis with current assessments of physical activity and current symptom severity. This methodological approach limits temporal interpretation and makes it difficult to determine whether behavioral adaptations reflected responses to initial symptom severity, changes over time, or factors potentially influencing current symptoms.

Second, the fibromyalgia diagnosis was self-reported, which may introduce misclassification bias. Third, the use of a non-validated questionnaire represents a limitation; however, the instrument was specifically designed to capture directional behavioral changes following diagnosis, which are not directly assessed by standard validated tools. Additionally, the coding strategy combined “no change” and “no prior consumption” within the same category, which may have reduced behavioral granularity and potentially obscured differences between participants with stable behaviors and those with no prior exposure to specific dietary items.

In addition, lifestyle and dietary changes were self-reported and may be subject to recall bias, particularly among participants with longer disease duration. Physical activity was assessed using a simplified classification and should therefore be interpreted as a general indicator of behavioral tendencies rather than a precise measure. Finally, the study population was predominantly female, which may limit the generalizability of the findings.

Despite these limitations, the study provides a novel data-driven perspective on real-world behavioral adaptation in fibromyalgia, highlighting the presence of structured patterns of lifestyle modification following diagnosis.

## 5. Conclusions

This study provides a data-driven perspective on post-diagnostic lifestyle adaptations in patients with fibromyalgia and suggests that behavioral changes may form structured and interrelated patterns rather than occur in isolation. Three distinct adaptation profiles—minimal, selective, and comprehensive—were identified, reflecting potential heterogeneity in patient-driven responses following diagnosis.

No association was observed between the extent of lifestyle modification and symptom severity. These findings should be interpreted in the context of the study’s exploratory and cross-sectional design.

Overall, the findings highlight the complexity of real-world behavioral adaptation in fibromyalgia and support the need for longitudinal and phenotype-oriented research to better understand the role of lifestyle factors in disease management.

## Figures and Tables

**Table 1:** Baseline characteristics of patients with fibromyalgia.

Parameters	
Sex (N)	88
Female (%)	84 (95.5%)
Male (%)	4 (4.5%)
Age (years), mean ± SD	46.9 ± 11.3
BMI (kg/m^2^), mean ± SD	25.6 ± 4.6
Time since diagnosis	N (%)
<3 years	41 (46.6)
3–6 years	21 (23.9)
>6 years	26 (29.5)
WPI, mean ± SD	16.68 ± 7.04
SSS, mean ± SD	6.09 ± 1.79

BMI—body mass index; WPI—Widespread Pain Index; SSS—Symptom Severity Scale; SD—standard deviation.

**Table 2:** Types of physical activity reported by patients with fibromyalgia (N = 88).

Type of Physical Activity	N	%
Walking	67	76.1
Yoga	26	29.5
Cycling	14	15.9
Nordic walking	10	11.4
Swimming	10	8.0

**Table 3:** Significant Spearman correlations between dietary behavior changes following fibromyalgia diagnosis; N = 88 (*p* < 0.05, |ρ| ≥ 0.25).

Variable 1	Variable 2	Spearman’s p	*p*-Value	Interpretation
Vegetables ↑	Water ↑	0.32	0.01	moderate positive
Sugar-sweetened beverages (cola) ↓	Alcohol ↓	0.41	<0.001	moderate positive
Vegetables ↑	Cola ↓	0.28	0.02	weak-to-moderate positive
Vegetables ↑	Alcohol ↓	0.36	0.01	weak-to-moderate positive

Only statistically significant correlations meeting the predefined threshold (*p* < 0.05 and |ρ| ≥ 0.25) are presented. Spearman’s rank correlation coefficients were calculated using ordinally coded behavioral change variables (+1 = increase, 0 = no change/no prior consumption, −1 = decrease), ↑ indicates an increase; ↓ indicates a decrease.

**Table 4:** Characteristics of dietary adaptation clusters.

Cluster	Adaptation Pattern	N (%)	Main Behavioral Characteristics	Mean WPI + SSS ± SD
1	Comprehensive health-oriented pattern	14 (15.9)	Increased vegetables, fruits, whole grains, and water intake; reduced meat, alcohol, and sugar-sweetened beverage consumption	23.21 ± 4.12
2	Selective dietary modification pattern	27 (30.7)	Partial increase in plant-based foods and selective reduction in specific dietary products	22.92 ± 3.87
3	Minimal adaptation pattern	47 (53.4)	Limited or inconsistent dietary and lifestyle modifications	22.80 ± 4.55

Differences between clusters were assessed using the Kruskal–Wallis test. No statistically significant differences in symptom severity were observed between clusters (*p* = 0.53).

## Data Availability

The original contributions presented in this study are included in the article. Further inquiries can be directed to the corresponding author.
